# Psychometric functioning, measurement invariance, and external associations of the Relationship Assessment Scale in a sample of Polish Adults

**DOI:** 10.1038/s41598-022-26653-6

**Published:** 2022-12-22

**Authors:** Katarzyna Adamczyk, Paweł Kleka, Monika Frydrychowicz

**Affiliations:** Faculty of Psychology and Cognitive Science, Adam Mickiewicz University, ul. A. Szamarzewskiego 89/AB, 60-568 Poznań, Poland

**Keywords:** Psychology, Health care

## Abstract

The current article reports data from three Polish samples to examine the Relationship Assessment Scale (RAS) with respect to its unidimensionality, invariance across countries, gender, formal and informal relationships, degree of precision (or information) across latent levels of relationship satisfaction, and the functioning of individual items. The analyses of the data from the reference sample (*n* = 733) confirmed a clear 1-factor structure of the RAS-PL and good internal consistency. Configural, metric, and scalar invariance for countries (Poland, Hungary, USA), gender (women and men) and relationship types (formal and informal relationships) were achieved. Item Response Theory Analysis (IRT) suggested that the RAS-PL assesses relationship satisfaction most reliably at low to average levels. Analyses of the data from validation samples (*n* = 203 and *n* = 209) confirmed the convergent and divergent validity by weak, medium, and large correlations of the RAS-PL with measures of other theoretically related constructs. Concurrent criterion validity was demonstrated by a strong positive correlation between the RAS-PL and the intent to continue the current relationship. This investigation provides considerable psychometric information about the items and scale of the RAS-PL.

## Introduction

Relationship satisfaction is associated with various outcomes for both individuals' and families' well-being^1,2^. Therefore, “[…] the importance of knowing more about how to measure adjustment in intimate partnerships”^3^, p 1029 as well as the psychometric properties of the research instruments used to measure relationship satisfaction is particularly crucial^2,4^. Among multiple research instruments designed to measure relationship satisfaction, the 7-item Relationship Assessment Scale (RAS)^5,6^ is one of the most widely and frequently used tools to assess satisfaction with marital and nonmarital relationships^7,8^ and has been adapted for the assessment of satisfaction with other types of relationships, such as relationships with parents, friends, and other types of relatives^9^.

Many psychometric aspects of the RAS have been investigated in prior research, e.g., unidimensionality, convergent, divergent, and discriminant validity, and test–retest stability ^1,5,6^_._ However, the measurement invariance of the RAS across diverse groups and the item and test properties have not yet been examined within an Item Response Theory (IRT) framework, except in the study by Funk and Rogge^2^. These authors employed a principal-component analysis (PCA) and IRT analysis to examine a pool of 180 items originating from eight tools measuring relationship satisfaction (including the RAS) to assess these items' precision in assessing satisfaction to create a new tool that assesses relationship satisfaction^7^. Although the primary aim of Funk and Rogge's analysis was not to provide comprehensive information on the properties of the RAS, the authors showed that the RAS items are globally worded and relatively homogeneous (e.g., RAS item no. 2) and provide relatively high amounts of information to assess satisfaction^7^. In a recent Hungarian study, Fülöp et al. attempted to develop and validate a single-item version of the original RAS^8^. In two studies utilizing samples of Hungarian individuals, Fülöp et al. employed structural equation modeling (SEM) to explore the linkages between relationship satisfaction measured by the full 7-item RAS and the 1-item RAS and other proximal and distal psychological constructs^8^.

## Correlates and outcomes of relationship satisfaction

Prior research has documented the link between high satisfaction, higher levels of mental and physical health, personal and family well-being among married individuals^2^, and greater emotional and psychological well-being^10^. Relationship satisfaction is related to greater support and less conflict^11^, and marital satisfaction is negatively related to impulsivity^12^. High relationship satisfaction is related to satisfaction with relationship status, i.e., satisfaction with having a partner^13^, while lower satisfaction is linked to problems in the mental health domain, including depression and loneliness^10,14–16^. Poorer marital functioning and low marital satisfaction are also related to work stress^17^. For instance, low marital satisfaction is associated with a lack of work enjoyment and high work involvement among female professionals^18^. At the same time, other studies have revealed that workaholism might not be related to adverse relationship outcomes, including lower relationship satisfaction^19^.

Finally, high marital satisfaction was found to be related to a lower likelihood of divorce. Individuals in low-satisfaction marriages are approximately twice as likely as those in high-satisfaction marriages to divorce^20^. Notably, relationship satisfaction measured by the employment of the RAS in comparison to the DAS seemed to be a more effective discriminating factor between couples who continued their relationship and couples who broke up, making the RAS a useful tool in detecting couples who may be “at risk” of ending a relationship^6^.

## Relationship satisfaction across countries

Cultural variation affects marital and relationship satisfaction^21^. Tai et al. demonstrated several differences in relationship satisfaction among diverse countries and showed that Australian and French individuals experienced lower satisfaction than German individuals^22^. Wiik et al. showed that cohabitation, in contrast to marriage, was related to lower quality across eight European countries^23^. The cohabitation gap in relationship satisfaction was lower in countries with a higher prevalence of cohabitation^23^. In past research, the measurement invariance of relationship quality assessed in terms of relationship satisfaction, commitment, intimacy, and trust across four diverse national samples (United States, Canada, Indonesia, and China) was tested by Gere and MacDonald^24^. The authors demonstrated that four scales measuring the above-indicated constructs met the criteria for weak measurement equivalence; however, three of them did not meet the criteria for strong measurement equivalence^24^.

## Relationship satisfaction and gender

Early studies^25^ and recent cross-cultural research^26^ revealed that men experienced higher marital satisfaction than women; however, in other studies, no gender differences were observed^2,26^. A meta-analysis of 226 studies revealed that for nonclinical community-based samples, the effect size showed a lack of significant gender differences in marital satisfaction^27^. Nevertheless, the role of gender in marital satisfaction may differ across diverse cultures as a function of culturally specific factors such as sex roles or sexual egalitarianism^28^. Although a substantial body of research has focused on sex and gender differences in relationship satisfaction and has found mixed results, we are unaware of prior research that has tested the gender invariance of the RAS.

## Relationship satisfaction across intimate relationships

A more recent comparison between different union types showed that married couples who premaritally cohabited and cohabitating couples who planned to marry did not differ with regard to relationship quality^26^. As a result of changes in the contemporary context of intimate relationships, whether the RAS assesses the same construct of relationship satisfaction within diverse types of relationships has been questioned. This question was proposed approximately 9 years ago by Graham et al., who performed a reliability-generalization meta-analysis of seven measures of relationship satisfaction, including the RAS^1^. However, these authors did not establish the measurement invariance of the relationship satisfaction construct across diverse types of relationships, such as nonmarital, engaged, and marital relationships^1^. Therefore, there is little knowledge of the psychometric properties of the original RAS in terms of its measurement invariance across different romantic relationships.

## The current study

Although a Polish translation of the RAS exists (RAS-PL)^29^ and has been utilized by Polish researchers, the psychometric properties of the RAS have yet to be subjected to a solid and comprehensive investigation with a Polish sample. Thus, the primary aim of the current study was to assess the psychometric functioning of the existing Polish translation of the RAS30 to provide additional information on the psychometric properties of the RAS by assessing its measurement invariance across countries (Poland, Hungary, the USA), genders (women vs. men) and relationship type groups (marital and nonmarital relationships) and its general functioning based on an IRT analysis.

To establish country invariance, we compared three national samples from Poland (reference sample; *n* = 733), Hungary (*n* = 703), and the USA (*n* = 200). We chose Hungary and the USA since (a) the original RAS was developed, validated and subsequently used in samples originating from the USA, and (b) different marital and relational contexts characterize Poland and Hungary compared to the USA. Moreover, Poland and Hungary differ from each other. For instance, in Hungary, cohabitation was not prevalent until the late 1980s, but cohabitation and living apart together relationships (LAT) have become more common, whereas the marriage rate is declining^30,31^. Hungary falls in the mid-range of countries with the highest proportion of married people (Romania and Poland) and the lowest proportion (Norway, Austria, the Netherlands, and Belgium)^31^. To test whether the RAS is invariant, or unbiased, in how it assesses relationship satisfaction across countries, we utilized (a) data collected in the scope of the current investigation in Poland, (b) data collected in Hungary by Fülöp et al.^8^ and (c) the subset of data collected in the USA in the scope of another investigation conducted and described in detail by Adamczyk et al.^32,33^^.^

Some previous studies provided a theoretical basis to expect gender differences in relationship satisfaction^21,25^. However, given mixed findings in this domain, we considered testing gender invariance an essential and current task. Therefore, to establish invariance across gender, we compared women and men in the Polish sample (*n* = 733) and additional tests within Hungary and the USA. Invariance across gender was tested separately for the Polish, Hungarian, and U.S. samples since prior country invariance might not have been assumed. Analogically, establishing measurement invariance across contemporary relationship types of different levels of commitment (i.e., nonmarital through engaged to marital relationships) is also justified. Previous research has revealed differences in the quality of cohabitating and marital relationships, with more recent studies demonstrating the lack of these differences^26^. Thus, to test invariance across relationship types, for this analysis with the Polish reference sample (*n* = 733), we combined participants in marital and engaged relationships into one group termed formal relationships (*n* = 322). In contrast, individuals who did not indicate being in a marital or engaged relationship (*n* = 401) were classified as a group of informal relationships. Due to the varying size of the formal and informal groups in the Hungarian and U.S. samples, we could not provide a robust invariance test for the relationship type groups in these two countries; therefore, one model is for both nonmarital relationship groups.

Third, to provide detailed information about the psychometric properties of the Polish RAS, the current study drew on Item Response Theory (IRT) methods. The prominent feature of IRT is the assumption that item responses are a function of the characteristics of the properties of the item (item parameters) and the characteristics of an individual (person parameters)^34,35^. Regarding the deficit of the employment of IRT methods for the original RAS, using IRT analysis to identify which Polish RAS items are most closely related to the latent construct of relationship satisfaction may contribute to a better understanding of this construct.

The integration of IRT/MIRT and CFA analyses is recommended in the literature because these two classes of analyses provide different indicators of psychometric performance; therefore, they can jointly provide complementary information pertaining to the dimensionality of instruments and item functioning^34–36^. The simultaneous use of IRT/MIRT and CFA analyses offers several benefits. Specifically, (1) CFA provides the assessment of item fit in terms of error variances, communalities, and factor loadings, whereas in IRT, item fit is performed by unweighted (outfit) and weighted (infit) mean square errors^37^; (2) in CFA, the link between the indicator and latent variable is limited to a linear relationship, whereas in IRT, it can be nonlinear^38^; (3) in CFA, the factor loading is used to indicate the relationship between the indicator and the latent variable across all levels of the latent variable, whereas in IRT, this relationship is provided across the range of possible values for the latent variable^38^. In IRT, indices of item information functions (IIF) and test information functions (TIF) can be established; while these indices are available in CFA, they are conditional on the other items on the measure^35^; and (4) a broad spectrum of indices assessing model fit is available in CFA, whereas in IRT, only the χ^2^ deviance statistic is available to assess model fit^35^.

Furthermore, convergent, divergent, and concurrent criterion validity were assessed by testing the associations between the Polish RAS and a series of psychological constructs. Specifically, the convergent and divergent validity of the Polish RAS were examined in validation sample 1 (N = 203) by testing the associations between the Polish RAS and measures of related constructs, i.e., the Couples Satisfaction Index (CSI-4)^7^ assessing relationship satisfaction, the Relationship Satisfaction Status Scale (ReSta)^13^ assessing satisfaction with relationship status, and the Quality of Relationships Inventory (QRI)^39^ measuring relational depth, support and conflict, impulsive behavior (SUPPS-P)^40^, work enjoyment and work involvement (WorkBAT)^41^ and fear of being single (FBSS)^42^.

To provide additional information on the Polish RAS’s convergent and divergent validity of the Polish RAS, we utilized the subset of Polish data collected in the scope of another project by Adamczyk et al.^32,33^. This subset of the data (validation sample 2; N = 209) allowed us to assess the associations between the Polish RAS and the Mental Health Continuum-Short Form^43^, which assesses emotional and psychological well-being, the SF-12v2^44^, which measures physical and mental health, the Centre for Epidemiological Studies-Depression Scale (CES-D)^45^, which assesses depressive symptoms, and the Social and Emotional Loneliness Scale for Adults SELSA (SELSA-S)^46^, which measures romantic loneliness.

Finally, concurrent criterion validity was investigated by the associations between the Polish RAS and intent to continue the current relationship. Based on prior research, we expected that relationship satisfaction measured by the Polish RAS (a) would be positively associated with relationship satisfaction measured by the CSI-4, satisfaction with relationship status (ReSta), relationship depth and support (QRI), mental and physical health (the SF-12v2), and emotional and psychological well-being (MHC-SF); (b) would be negatively related to relationship conflict (QRI), impulsive behavior (SUPPS-P), depression (CES-D), romantic loneliness (SELSA-S), work enjoyment and work involvement (WorkBAT); and (c) would not be or would be weakly associated with fear of being single (FBSS). We hypothesized that higher relationship satisfaction would be related to higher intent to continue the current relationship with respect to concurrent criterion validity.

## Method

The study protocol was approved by the Ethics Committee for Research with People as Study Participants at the Faculty of Psychology and Cognitive Science, Adam Mickiewicz University in Poznań, Poland (Decision no. 1/01/2021). The study protocol was performed in accordance with the ethical standards of the Ethics Committee for Research with People as Study Participants at the Faculty of Psychology and Cognitive Science, Adam Mickiewicz University in Poznań and with the 1964 Helsinki Declaration and its later amendments or comparable ethical standards. All respondents consented in a written manner before beginning the survey. Informed consent was obtained to publish the information in an online open-access publication.

Participants were compensated for their participation in the study (i.e., participants might win vouchers worth 15 and 20 PLN to a Polish online store). The data in the reference and validation sample 1 were obtained through an online survey using Google Forms between March 15 and April 29, 2021. Participants were recruited through advertisements posted on Facebook that included a description of the study's goals, informed consent, and a link to the survey. The survey took approximately 15 min to complete.

The minimal sample size of the validation study was determined based on an a priori power calculation (https://sample-size.net/correlation-sample-size/). Specifically, to detect small-sized correlation coefficients (0.20) with sufficient statistical power (0.80), the study would require at least 194 respondents. Furthermore, since our analyses employed the IRT-based methodology that requires a large sample size (N > 500), we allowed a larger reference sample size to be recruited to meet the requirements of IRT analysis.

### Samples

#### Reference Polish sample

In total, 805 Polish respondents began the study; however, two did not consent to participate, and 30 were single and did not meet the criterion of being in a relationship. Hence, the final sample consisted of 733 participants, including 416 women (56.80%) and 317 men (43.20%), with a mean age of 32.70 years (ranging from 18 to 75 years). For relationship type, 401 participants (54.70%) were in nonmarital relationships, 121 were engaged (16.50%), and 211 were in marital relationships (28.80%). Further characteristics of the sample, including place of residence, highest education level, having a child/children, living with a partner, and duration of the relationship, are provided in Table S1 in the online supplementary materials.

#### Validation samples

The first validation sample consisted of 203 Polish adults [161 women (79.30%), 42 men (20.70%), *M*_*age*_ = 31.14 *(SD* = 7.56), range 19–54 years]. For relationship type, 123 individuals were in nonmarital relationships (60.60%), 45 were engaged (22.20%), and 35 were in marital relationships (17.20%). Detailed characteristics of the sample are included in Table S1 in the online supplementary materials.

The second validation sample came from a study on relationship status and mental health among Polish and U.S. young adults^32,33^. Validation sample 2 included 209 Polish adults (137 females, 65.60%; 72 males, 34.40%; *M*_*age*_ = 24.91 *SD* = 4.83; range 19–46 years). Regarding relationship type, 37 individuals were married (17.70%), 140 individuals were engaged (67.00%), and 32 individuals were in nonmarital relationships (15.30%). The mean duration of a relationship was 3.37 years (*SD* = 3.50). Detailed characteristics of the sample are included in Table S1 in the online supplementary materials. A description of the study from which sample 2 was derived is provided in the papers by Adamczyk et al.^32,33^.

The Hungarian validation sample came from the study by Fülöp et al.^8^. The full sample contained 703 participants who were aged 18 to 64 years (*M* = 25.61, *SD* = 8.00), including 360 females (51.20%). Among Hungarian participants, 67 individuals (9.50%) had casual relationships, 349 individuals (49.60%) were in a nonmarital relationship but did not live with a partner, 180 individuals (25.60%) were in a nonmarital relationship and lived with a partner, 49 individuals (7.00%) were engaged, and 58 individuals (8.30%) were married. The mean duration of a relationship was 2.93 years (*SD* = 4.13). Detailed characteristics of the Hungarian participants are provided in the paper by Fülöp et al.^9^.

The fourth U.S. sample consisted of participants enrolled in a study of relationship status and mental health among Polish and U.S. young adults^36,42^. The subset of data utilized in the current study involved data from 200 U.S. participants who were in relationships, including 139 women (69.50%) and 61 men (30.50%) aged 19–40 years (*M* = 21.94, *SD* = 3.19). Among the U.S. participants, 185 were in nonmarital relationships (92.50%) and 15 were engaged (7.50%). The mean duration of a relationship was 2.18 years (*SD* = 2.74). Most participants had graduated from high school (*n* = 72, 36%). The detailed characteristics of the U.S. participants are provided in Table S1 in the online supplementary materials and the papers by Adamczyk et al.^32,33^.

## Materials

### The Relationship Assessment Scale

The Relationship Assessment Scale (RAS)^5,6^ in the Polish translation by Monfort et al.^29^ was completed by participants in the reference sample and validation sample 1. The RAS consists of 7 items assessing global relationship satisfaction (e.g., "How much do you love your partner?"). It was created by utilizing a sample of dating university students. Each item is rated on a 5-point Likert scale from 1 (i.e., *not satisfied*) to 5 (i.e., *very satisfied*). Higher scores indicate higher relationship satisfaction. In the current study, we computed a mean score. The internal consistency of the Polish RAS is provided in Table 1. The omega coefficient for the RAS was 0.90 and 0.86 in the Hungarian and U.S. samples, respectively.

**Table 1:** Descriptive and reliability statistics for the Polish RAS.

	*M (SD)*	Cronbach’s alpha	Omega total	Range of inter-item correlations	Mean inter-item correlation^a^
Reference sample (*n* = 733)	4.16 (0.72)	0.89	0.89	0.29–0.78	0.56
Validation sample 1 (*n* = 203)	4.03 (0.61)	0.81	0.81	0.30–0.56	0.43
Validation sample 2 (*n* = 209)	4.11 (0.74)	0.87	0.88	0.15–0.77	0.52

*RAS* Relationship Assessment Scale.

^a^The mean inter-item correlation of the original RAS was determined to be 0.49 (Hendrick^6^).

### Demographic information

Respondents in the reference sample and validation sample 1 were asked to indicate their age (in years), the gender they identified with most (“male”, “female”, “other”), their sexual orientation (“heterosexual”, “homosexual”, “bisexual”, “do not know”), their place of residence (from “village” to “city > 500,000 “), their highest educational level obtained (from “primary education” to “higher education”), relationship status (“single”, “partnered”), type of relationship (“informal relationship”, “engaged relationship”, “marriage”), living with a partner (“yes”, “no”), duration of living with a partner (in months), duration of the relationship (in months), having a child/children (“yes”, “no”), and intent to continue the current relationship rated on a 4-point Likert scale from 0 (*not at all*) to 3 (*very much*).

### Validation questionnaires

For validation, in validation sample 1, we used the Polish versions of the Couples Satisfaction Index (CSI-4)^47^, the Relationship Satisfaction Status Scale (ReSta)^10,13^, the Quality of Relationships Inventory (QRI)^39^, the Fear of Being Single Scale (FBSS)^42^, the Workaholism Battery^41^, and the Impulsive Behavior Scale Short Version (SUPPS-P)^40^. Participants in validation sample 2, from a study by Adamczyk et al.^33^, completed the Polish versions of the Mental Health Continuum-Short Form (MHC-SF)^43^, the Centre for Epidemiological Studies-Depression Scale (CES-D)^45^, the Social and Emotional Loneliness Scale for Adults SELSA (SELSA-S)^46^ and the SF-12v2^44^. For details regarding the validation questionnaires in validation samples 1 and 2, see the online supplementary materials.

### Data analysis

Data analysis proceeded in five steps.

The first step verified the 1-factor structure of the Polish RAS in Polish reference sample 1 using confirmatory factor analysis (CFA). Given that the RAS items are ordinal, normality was not assumed. The weighted least square mean and variance adjusted estimator was employed because it is recognized to be appropriate when the data are not normally distributed^48^. Based on recommendations provided by Whittaker^49^, CFI and TLI values ≥ 0.90 showed acceptable model fit, whereas RMSEA and SRMR values < 0.08 demonstrated good model fit. Due to deviations from the multivariate normality of the distribution, the correction proposed by Satorra-Bentler was applied^50^. The CFA was performed with the lavaan package^51^ in R version 4.2.1^52^.

The second step assessed the internal consistency of the Polish RAS scores in reference and validation samples 1 and 2 using both Cronbach’s alpha (α) and McDonald’s omega (ω) coefficients. Cronbach’s and McDonald's omega values of ≥ 0.70 and ≥ 0.80 were considered to demonstrate acceptable and good internal consistency, respectively^53,54^. Furthermore, the range of interitem correlations and the mean of each interitem correlation of the Polish RAS in the Polish samples 1–3 were also determined. Internal consistencies and descriptive statistics were conducted in SPSS 27.0 (IBM SPSS Statistics).

In the third step, we tested three levels of measurement invariance between the groups distinguished based on (1) the country of origin (Poland, Hungary, and the USA), (2) gender (women and men), and (3) type of relationship (marital and nonmarital relationships). First, we tested the weakest form of measurement invariance, configural invariance, which assumes that the latent constructs (i.e., the factors) exhibit the same dimensionality and that the indicators (i.e., the items) may be identically assigned to the latent constructs in the analyzed groups^55^. Establishing configural invariance is a prerequisite to performing unbiased measurement comparisons between groups^55^. To examine this type of invariance, we allowed all factor loadings and item thresholds to vary freely in each group.

Second, we examined the more restrictive form of measurement invariance, metric invariance (weak invariance), to determine whether the factor loadings were equivalent across samples^24,55^. To assess this type of invariance, the factor loadings were constrained to be equivalent across groups, whereas the item thresholds were allowed to vary freely^24^. The establishment of metric invariance allows for comparisons of structural relationships among latent constructs (e.g., correlation coefficients) between groups^55^.

Third, we assessed the scalar (strong) invariance to examine whether the item thresholds were equivalent across samples by constraining the item thresholds and factor loadings to be equivalent^24,25^. Confirming strong invariance allows for the assessment of the between-group differences in the constructs’ means^55^. We did not test strict invariance (the equivalence of the residual variance of the items) because this level of invariance is considered to be highly restrictive and is rarely established using real data^24^. Passing or failing the metric and scalar invariance testing was determined by the size of the change in CFI calculated by subtracting the CFI of the less-constrained model (e.g., the configural invariance model) from the more-constrained model (e.g., the metric invariance model). Invariance was supported if the change in CFI was below 0.010 and an increase in RMSEA was below 0.015^56^. When invariance was not supported, we identified the specific items that created variance across groups.

In the fourth step, the Item Response Theory (IRT) analysis with the generalized partial credit model (GPCM) scaling method was used to analyze the reliability of individual test items of the Polish RAS and for additional testing of the Hungarian RAS. Information function values and information curves were presented. The curves showed the amount of information provided for a given feature level, which was adequate for the precision of the position in the area of a given feature level^57^. IRT analyses were conducted using the ltm package^57^ in R version 4.0.2^52^.

In the last step, convergent, divergent, and concurrent criterion validity were assessed in validation samples 1 and 2 by computing Pearson correlations (two-tailed) between the Polish RAS scores and the scores on self-report measures of theoretically related constructs (i.e., CSI-4, ReSta, QRI, FBSS, SUPPS-P, WorkBAT, MHC-SF, CES-D, SELSA-S, and the SF-12v2). In turn, to assess the criterion validity of the Polish RAS in the Polish validation sample 1, a hierarchical regression analysis was performed to test whether the RAS score (dependent variable) was predictive of the intent to continue the current relationship. These analyses were conducted in SPSS 27.0 (IBM SPSS Statistics).

## Results

### Factor structure

**Figure 1:**
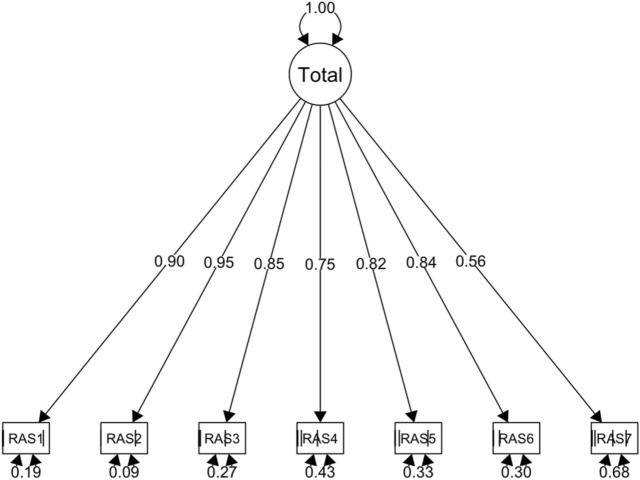
The path diagram of the one-factor model of the Polish RAS in the Polish reference sample (N = 733). *RAS* Relationship Assessment Scale.

The results of a CFA analysis in the Polish reference sample (*n* = 733) showed that the initial one-factor model fit the data well (χ^2^(14) = 109.252, *p* < 0.001, CFI = 0.998, TLI = 0.998, RMSEA = 0.058, see Table 2). The path diagram of the model is presented in Fig. 1. All loadings for the seven items were statistically significant (*p* < 0.001). The loadings of each item on the relationship satisfaction latent construct in the 1-factor model are reported in Table S2 in the online supplementary materials.

**Table 2:** Model fit for multiple group models and measurement invariance comparisons across countries.

Model RAS (7 items)	chi^2^ (df)	RMSEA [90% CI]	SRMR	CFI	TLI	Δ chi^2^ S-B	Δ CFI	Δ RMSEA	Decision
One factor pooled sample (*n* = 1635)	187.656*** (14)	0.052 [0.046, 0.059]	0.034	0.998	0.997	–	–	–	Pass
Country									
Baseline Poland sample (*n* = 733)	109.252*** (14)	0.058 [0.048, 0.069]	0.032	0.998	0.998	–	–	–	Pass
Baseline Hungary sample (*n* = 703)	119.282*** (14)	0.063 [0.053, 0.074]	0.037	0.998	0.996	–	–	–	Pass
Baseline U.S. sample (*n* = 200)	57.521*** (14)	0.083 [0.061, 0.106]	0.049	0.997	0.995	–	–	–	Pass
Configural invariance	279.611*** (42)	0.064 [0.057, 0.071]	0.036	0.998	0.997	–	–	–	Pass
Metric invariance	357.430*** (54)	0.081 [0.073, 0.089]	0.045	0.996	0.995	79.586**(12)	− 0.002	0.017	Pass*
Scalar invariance	437.500*** (94)	0.063 [0.057, 0.069]	0.057	0.995	0.997	57.142*(40)	− 0.001	− 0.018	Pass
Country, Monte Carlo sampling (*n* = 200 for each group, number of samples = 100)									
Configural invariance	240.057*** (42)	0.077 [0.068, 0.087]	0.041	0.997	0.996	–	–	–	Pass
Metric invariance	330.785*** (54)	0.100 [0.090, 0.111]	0.054	0.994	0.993	90.72***(12)	− 0.003	0.023	Pass*
Scalar invariance	245.001*** (94)	0.077 [0.068, 0.086]	0.116	0.994	0.996	5.91 (40)	0	− 0.023	Pass

Differences between groups are on the loadings of item RAS1: λ PL = 0.887 ± 0.09, λ HU = 0.889 ± 0.09, λ US = 0.865 ± 0.09. After relax this parameter constraint invariance was achieved: Δ RMSEA = 0.009 < 0.015 and Δ CFI = -0.001 < 0.010. * based on SRMR < 0.06, because CFI and RMSEA indices are inconsistent.

*CFI* comparative fit index, *TLI* Tucker–Lewis index, *RMSEA* root mean square error of approximation, *CI* confidence interval, *df* degrees of freedom, *SRMR* squared root mean residuals, *S-B* Satorra–Bentler correction, *Δ* comparisons of nested model: configural-metric, metric-scalar.

### Internal consistency

Table 1 shows the Cronbach’s alphas and omega coefficients for the RAS in the reference and validation samples, the range of interitem correlations, and the mean of each interitem correlation. Cronbach’s alphas showed good internal consistency (0.81–0.89 in three samples), and the McDonald's omega total values were identical to Cronbach’s alpha (except for sample 2, in which the omega coefficient was higher than Cronbach’s alpha). The seven items of the RAS were all positively correlated, with a mean interitem correlation of 0.56, 0.43, and 0.52 in Studies 1, 2, and 3, respectively (the interitem correlations in three studies ranged from 0.15 to 0.78).

### Measurement invariance

The results of measurement invariance analyses across the country, gender, and relationship type groups are shown in Tables 2, 3, and 4. Tables 2, 3 and 4 also include the values of Standardized Root Mean Squared Error (SRMR), which has been shown to perform better than the Root Mean Squared Error of Approximation (RMSEA) when data are estimated as categorical in nature^58^ as well as for models with small *df*, for which SRMR is recommended^59^.

**Table 3:** Model fit for multiple group by gender models and measurement invariance comparisons.

Model	chi^2^ (df)	RMSEA [90% CI]	SRMR	CFI	TLI	χ^2^ S-B (df)	Δ CFI	Δ RMSEA	Decision
Baseline model for women (*n* = 915)	134.310***(14)	0.060 [0.051, 0.069]	0.040	0.998	0.997	–	–	–	Pass
Baseline model for men (*n* = 720)	67.609***(14)	0.043 [0.033, 0.054]	0.029	0.999	0.998	–	–	–	Pass
Poland (women, *n* = 416; men, *n* = 317)									
Configural invariance	132.83***(28)	0.063 [0.052, 0.073]	0.035	0.998	0.997	–	–	–	
Metric invariance	112.03***(34)	0.059 [0.047, 0.071]	0.039	0.998	0.998	8.51 (6)	0	− 0.004	Pass
Scalar invariance	148.55***(54)	0.050 [0.041, 0.060]	0.042	0.998	0.9958	33.26*(20)	0	− 0.009	Pass
Hungary (women, *n* = 360; men, *n* = 343)									
Configural invariance	136.14***(28)	0.065 [0.054, 0.076]	0.039	0.998	0.996	–	–	–	
Metric invariance	118.32***(34)	0.063 [0.051, 0.075]	0.042	0.997	0.997	10.15 (6)	− 0.001	− 0.002	Pass
Scalar invariance	169.31***(54)	0.057 [0.048, 0.067]	0.039	0.996	0.997	49.63***(20)	− 0.001	− 0.006	Pass
USA (women, *n* = 139; men, *n* = 60)									
Configural invariance	69.392***(28)	0.083 [0.059, 0.108]	0.050	0.997	0.996	–	–	–	
Metric invariance	63.419***(34)	0.076 [0.046, 0.105]	0.057	0.997	0.996	10.695 (6)	0.008	− 0.013	Pass
Scalar invariance	105.049***(54)	0.076 [0.054, 0.097]	0.083	0.995	0.996	8.099 (6)	0.001	0.001	Pass

*CFI* comparative fit index, *TLI* Tucker–Lewis index, *RMSEA* root mean square error of approximation, *CI* confidence interval, *df* degrees of freedom, *SRMR* squared root mean residuals; *S–B* Satorra–Bentler correction, Δ comparisons of nested model: configural-metric, metric-scalar. In the U.S. sample, one outlier was excluded due to Cook’s distance above 1.

*p < 0.05, ***p < 0.001.

**Table 4:** Model Fit for Multiple Groups by the Relationship Type Models and Measurement Invariance Comparisons.

Model	chi^2^ (df)	RMSEA [90% CI]	SRMR	CFI	TLI	Δ χ^2^ S-B	Δ CFI	Δ RMSEA	Decision
Baseline model for informal relationships (*n* = 1181)	168.49*** (14)	0.059 [0.051, 0.067]	0.037	0.998	0.997	–	–	–	Pass
Baseline for formal relationships (*n* = 454)	29.078** (14)	0.030 [0.0, 0.045]	0.036	0.999	0.999	–	–	–	Pass
Poland (informal relationships, *n* = 401; formal relationships, *n* = 332)									
Configural invariance	126.78*** (28)	0.061 [0.051, 0.072]	0.034	0.998	0.997	–	–	–	Pass
Metric invariance	95.52*** (34)	0.053 [040, 0.065]	0.034	0.998	0.998	3.33 (6)	0	–0.008	Pass
Scalar invariance	118.12*** (54)	0.041 [0.031, 0.052]	0.093	0.998	0.999	18.26 (20)	0	–0.012	Pass
Hungary (informal relationships, *n* = 596; formal relationships, *n* = 107)									
1 group model	119.282*** (14)	0.063 [0.053, 0.074]	0.042	0.998	0.996	–	–	–	Pass
U.S. (informal relationships, *n* = 184)									
1 group model	57.52** (14)	0.083 [0.061, 0.106]	0.055	0.997	0.995	–	–	–	Pass

*CFI* comparative fit index, *TLI* Tucker–Lewis index, *RMSEA* root mean square error of approximation, *CI* confidence interval, *df* degrees of freedom, *SRMR* squared root mean residuals,* S–B* Satorra–Bentler correction, Δ comparisons of nested model: configural-metric, metric-scalar.

*p < 0.05, **p < 0.01, ***p < 0.001.

#### Country

The invariance of the 1-factor model was first tested across countries. Baseline measurement models were tested for the Polish, Hungarian, and U.S. samples separately and fit the data adequately, Poland: χ^2^(14) = 109.252, *p* < 0.001, CFI = 0.998, TLI = 0.998, RMSEA = 0.058, 90% CI [0.048, 0.069], Hungary: χ^2^(14) = 119.282, *p* < 0.001, CFI = 0.998, TLI = 0.996, RMSEA = 0.063, 90% CI [0.053, 0.074], U.S.: χ^2^(14) = 57.521, *p* < 0.001, CFI = 0.997, TLI = 0.995, RMSEA = 0.083, 90% CI [0.061, 0.106]. The configural invariance model was then tested and fit the data adequately, indicating that the factor structure was invariant across countries (see Table 2 for configural model fit statistics).

Next, the metric (weak) invariance model, which constrained all factor loadings to invariance, was tested and compared with the configural invariance model. This model fit the data adequately according to the Δχ^2^ and SRMR because the threshold for Δ RMSEA was slightly exceeded (see Table 2). Finally, the scalar (strong) invariance model was tested and compared with the metric invariance model, which constrained all item thresholds to invariance. This model fit well to the data. A decrease in CFI greater than 0.01 and an increase in RMSEA greater than 0.015 were not observed in this case (see Table 2).

Because the U.S. sample was smaller and uneven group sizes might attenuate the sensitivity to detect noninvariant parameters in multiple group CFA analyses, we used Monte Carlo sampling techniques to test invariance across countries^60^. We randomly chose 200 participants from Poland and Hungary (the size of the U.S. sample) and computed all fit indices. We repeated this procedure 100 times. Generally, the results were the same as in the approach above; only metric (weak) invariance needed freed loadings to accomplish the level of fit. Model modification indices were examined to identify whether particular item loadings contributed significantly to the model misfit, and a partial metric model with freed loadings for item no. 1 ("How well does your partner meet your needs?") was tested. The loading for item 1 was lower in the U.S. sample than in the Polish sample and the Hungarian sample, suggesting that the assessment of the degree to which a current partner meets an individual's needs is more strongly related to relationship satisfaction in the Polish and Hungarian samples than among U.S. participants. The results are displayed in Table 2.

#### Gender

The invariance of the 1-factor model was tested across women and men in each of the countries. Baseline measurement models were fit for women and men separately and fit the data adequately (see Table 3 for model fit statistics for each group). For all samples, we achieved scalar (strong) invariance for gender.

#### Relationship types

Finally, the invariance of the 1-factor model was examined across relationship type groups only within the Polish reference sample, in which we achieved scalar (strong) invariance. The data fit the 1-factor model well for every level of the restrictions (see Table 4).

### Test and item properties

**Figure 2:**
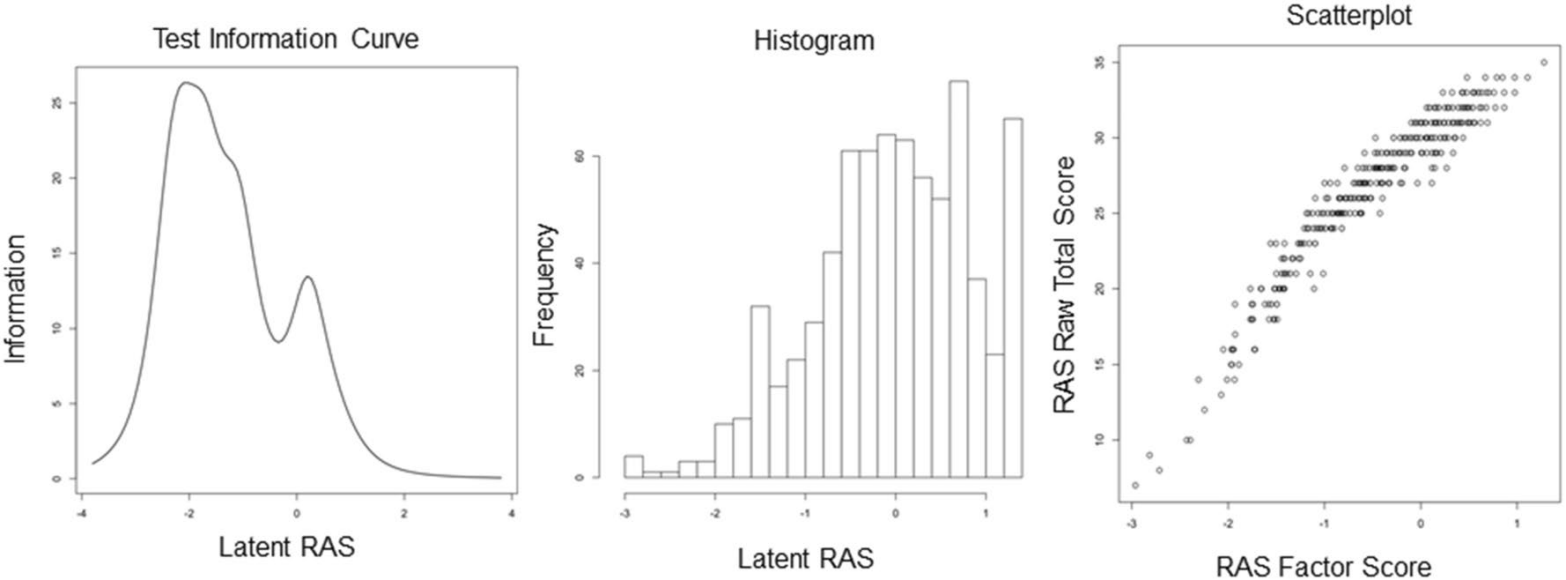
Test information curve for the Polish RAS, histogram of relationship satisfaction factor scores, and a scatterplot of raw v. factor scores for the Polish sample. *n* = 733. *RAS* Relationship Assessment Scale.

After meeting the IRT assumptions (unidimensionality: Velicer MAP has a minimum of 0.04 for 1 factor; local independence: pairwise correlation indices do not exceed 0.70), we determined the test and item properties of the Polish RAS (see Fig. 2 and Figure S1 in the online supplementary materials). In addition, to provide more robust results, we performed an IRT analysis using the data collected in Hungary (the detailed results for the Hungarian sample are provided in Figures S2 and S3 and Table S3 in the online supplementary materials).

As Fig. 2 shows, the total information curve indicates that the Polish RAS provides the most information or assesses with the slightest error, at approximately 2 SDs below the mean of latent relationship satisfaction. It provides very little information above 2 SDs. In the Hungarian sample, we determined that the RAS also provides the most information or assesses with the slightest error at approximately 2 SDs below the mean of latent relationship satisfaction (see Figure S2 in the online supplementary materials).

Figure 2 also presents the histogram that depicts the number of cases at all levels of latent relationship satisfaction. The scatterplot of factor scores against sum scores depicts the impact of weighting items by the discrimination parameters. At higher levels of latent relationship satisfaction (approximately 1 SD and above), the sum and factor scores were less strongly related. Analogically, in the Hungarian sample, with higher levels of latent relationship satisfaction (approximately 1 SD and above), the sum and factor scores were less strongly related.

The estimated item parameters (i.e., item discrimination and difficulty) are presented in Table 5. The Polish RAS items no 2 ("In general, how satisfied are you with your relationship?") had the highest discrimination, whereas item no. 7 had the lowest discrimination among the seven RAS items (see Table 5). The four sets of difficulty parameters were quite variable (1: − 1.920 to − 2.359; 2: − 1.847 to − 2.858; 3: − 0.936 to − 1.670; 4: − 0.841 to 0.561). These parameters were based on item thresholds and demarcate response categories 0 from 1, 1 from 2, 2 from 3, 3 from 4. Very high values are undesirable since they indicate that the measured construct is conceptually narrow. Good values should fit ranges of 0.5–2.5. The data in Table 5 show that the estimated parameters were of reasonable to very high magnitude except for items no. 1 and no. 2. Additionally, the estimated parameters for item no. 2 in the Hungarian sample were very high, whereas the parameters for item no. 1 were of acceptable magnitude (for the rest of the items, the parameters ranged from 0.0864 to 2.541; see Table S3 in the online supplement material).

**Table 5:** Item Difficulty Location and Thresholds For Informational Curves for the Polish RAS.

Items	*a*	SE	*b-1*	*b-2*	*b-3*	*b-4*	Information in range − 3, 3 SD	Proportion of information in range vs total information
RAS1	3.337	0.283	− 2.313	− 1.958	− 1.099	0.561	13.0223	0.9757
RAS2	5.259	0.589	− 2.319	− 1.687	− 1.069	0.209	20.8939	0.9932
RAS3	2.398	0.202	− 2.332	− 2.206	− 1.129	0.087	9.0794	0.9465
RAS4	1.254	0.110	− 2.064	− 1.669	− 1.172	− 0.419	4.5919	0.9157
RAS5	1.988	0.162	− 2.023	− 1.847	− 0.936	0.342	7.6465	0.9614
RAS6	1.982	0.187	− 2.359	− 2.074	− 1.670	− 0.841	7.3603	0.9283
RAS7	0.625	0.059	− 1.920	− 2.858	0.528	0.103	1.8790	0.7528

*n* = 733; *a* = discrimination parameter estimates in the RAS; *b*-1 = difficulty parameter estimates between response categories 0 and 1; *b*-2 = difficulty parameter estimates between response categories 1 and 2; *b*-3 = difficulty parameter estimates between response categories 2 and 3; *b*-4 = difficulty parameter estimates between response categories 3 and 4. Area under curve with range [− 3, 3] SD raw and percent, compared to total result of information function.

The inspection of the threshold parameters for the Polish RAS showed that most of the items were located to the left of the mean θ, indicating that most of the items were uninformative about individual differences at the range of the θ scale, where a distinction is made between moderately high levels of relationship satisfaction and very high levels. This is reflected in the test information function, which drops sharply above the mean θ in the Polish and Hungarian samples. From the inspection of the parameter estimates and test information functions for the Polish and Hungarian samples, it can be concluded that both scales discriminate best between individuals with low scores and average scores.

### Convergent, divergent, and criterion validity

The convergent, divergent, and criterion validity of the Polish RAS were assessed using data collected in the current validation sample 1 and validation sample 2^36,42^ (see Table 6).

**Table 6:** Convergent and divergent validity of the Polish Relationship Assessment Scale.

Validation constructs	Validation Sample 1 (*n* = 203)	Validation Sample 2 (*n* = 209)
CSI—relationship satisfaction	0.899***	–
ReSta—satisfaction with relationship status	0.842***	–
QRI—quality of relationship: depth of relationship	0.641***	–
QRI—quality of relationship: relationship support	0.685***	–
QRI—quality of relationship: relationship conflict	− 0.617***	–
SF-12v2—mental health component		0.409***
SF-12v2—physical component	–	0.217**
CES-D—depression	–	− 0.382***
MHC-SF—emotional well-being	–	0.407***
MHC-SF—psychological well-being	–	0.400***
SELSA-S—romantic loneliness	–	− 0.621**
SUPPS-P—impulsive behavior	− 0.142*	–
WorkBAT—work enjoyment	0.088	–
WorkBAt—work involvement/feeling driven to work	0.040	–
FBSS—fear of being single	− 0.099	–

Numbers represent correlations. *CSI* Couples Satisfaction Index, *ReSta* Relationship Satisfaction Status Scale, *QRI* Quality of Relationships Inventory, *SF-12v2* SF-12v2^®^ Health Survey, *CES-D* The Center for Epidemiologic Studies Depression Scale, *MHC-SF* Mental Health Continuum-Short Form, *SELSA-S* Social and Emotional Loneliness Scale for Adults-Short Form, *SUPPS-P* The Impulsive Behavior Scale Short Version, *WorkBAT* Workaholism Battery, *FBSS* Fear of Being Single Scale.

*p < 0.05, **p < 0.01, ***p < 0.001.

As demonstrated in Table 6, the results show weak to medium, large and very large correlations of the RAS score with all theoretically related constructs in both samples except for the constructs of workaholism and fear of being single. As expected, the RAS was positively correlated with relationship satisfaction assessed by a different instrument (CSI-4) and relationship quality measured in terms of relationship depth and support as well as positive outcomes such as mental health and emotional and psychological well-being. Furthermore, the RAS showed the expected opposite pattern in which the RAS was negatively correlated with relationship conflict, impulsive behavior, depressive symptoms, and romantic loneliness. Finally, relationship satisfaction was not correlated with two workaholism dimensions, i.e., work enjoyment (enjoying work to such a degree that it is difficult to stop working) and work involvement (the drive to work that involves the feeling of the obligation to work hard and thinking about work even when an individual wants to avoid it) or with the construct of fear of being single.

Concerning the assessment of the concurrent criterion validity of the Polish RAS, we performed a hierarchical regression analysis in which the intent to continue the current relationship was predicted from the demographic and relational variables entered in Step 1 (age, gender, type of a relationship, living vs. not living with a partner, duration of the relationship, duration of living with a partner, having children) and the RAS score in Step 2. The analysis revealed that in the last step, the only significant, positive predictors of the intent to continue the current relationship were living vs. not living with a partner (β = 0.16, *p* = 0.008) and the RAS score (β = 0.65, *p* < 0.001) as the strongest predictors. This model explained 38% of the variance to continue the current relationship. This means that individuals with higher relationship satisfaction and those living with a partner had higher intent to continue their current relationships. For the detailed results of the analysis, see Table S4 in the online supplementary materials.

## Discussion

The primary aim of the current investigation was to examine the Relationship Assessment Scale (RAS) with respect to its unidimensionality, invariance across countries, gender, formal and informal relationships, degree of precision (or information) across latent levels of relationship satisfaction, and the functioning of individual items.

Measurement invariance analysis using multiple-group CFA supported configural measurement invariance across countries (Poland, Hungary, USA), genders (women and men), and relationship types (formal and informal relationships). This means that the seven items of the RAS were representative of the relationship satisfaction construct across the analyzed groups. Our analyses also demonstrated that the RAS achieved metric (weak) measurement invariance across countries, genders, and relationship types. This means that each RAS item had equal loadings on the relationship satisfaction factor across the country, gender, and relationship type groups and each item had an equal contribution to the total score on the relationship satisfaction construct, which is the most critical criterion for establishing construct validity^61^. The establishment of metric (weak) measurement invariance also means that the RAS may be utilized in examining the associations between theoretical constructs, such as relationship satisfaction and other constructs^24^, in groups of Polish, Hungarian, and U.S. individuals, women and men, and groups of individuals in formal and informal relationships. We noted that item no. 1, "How well does your partner meet your needs?", did not have equal loadings on the satisfaction factor in the USA compared to Poland and Hungary. This finding implies that item no. 1 may be less relevant to relationship satisfaction in the USA compared to Poland and Hungary^62^.

Finally, we established full scalar (strong) invariance across countries, genders, and relationship type groups; that is, we demonstrated the equivalence of the factor loadings and the thresholds of items across the analyzed groups. The measurement invariance at the level of the thresholds indicates that items measure features of the trait that manifest to similar degrees in each of the countries, genders, and relationship types, contributing to similar probabilities of endorsement of the items^62^. This finding means that the RAS met the criterion of strong invariance, which allowed us to perform the mean-level differences across the analyzed groups^24^.

Concerning the characteristics of individual RAS items (specifically, item difficulty and discrimination) and the information (or precision) of the total RAS, the major finding is that the Polish and Hungarian versions of the RAS were found to provide the most accurate assessment, or the most information, at low levels of latent RAS. We demonstrated this in several ways, including the test information and individual item information curves, which peaked near the middle of latent RAS, and the strong positive relationship between the total raw scores and the model-estimated factor scores below the mean of latent RAS, which showed that differences between individuals at low levels are more meaningful than differences between individuals at high levels. For future research, this finding may imply the need to consider modifying the Likert scale employed in the RAS to increase the differentiation of options depicting the values at the high end of the scale (the end of the scale depicting higher levels of relationship satisfaction).

The low precision at higher levels of relationships suggests that the RAS is most suitable for assessing individuals with low to average relationship satisfaction levels. Although the instrument would be expected to have high precision across the entire trait continuum^63^, we also note a benefit of this precision in the low to average ranges of the relationship satisfaction continuum. Specifically, the RAS appears to be reliable in detecting individuals or couples who are in relationships with low satisfaction, which, as we have already noted, puts them at risk of negative individual and relational outcomes, including the risk of divorce/break up^64^, depression^14^ and loneliness^10,16^. For instance, most clients in couple therapy experience depression and/or problems in their relationships^15^.

To explain the precision of the RAS in low ranges of relationship satisfaction, we may refer to an analogy between the relationship satisfaction construct and the term "quasitrait" proposed by Reise and Waller^63^ regarding psychopathology constructs. Reise and Waller^63^ suggested that the peaked (in the severe trait range) information curve in IRT analyses of clinical scales arises from the unipolar character of the trait and is relevant only in one direction. Although we do not conceptualize relationship satisfaction as a psychopathology construct, we may tentatively consider the unipolar character of relationship satisfaction with reverse relevance involving low but not high ends of the continuum. Specifically, the relevant low end might indicate dissatisfaction with a relationship (negativity in a relationship), while the high end would not indicate relationship satisfaction (positivity in a relationship) but rather a lack of dissatisfaction with a relationship (the absence of negativity). As a result, if relationship satisfaction were considered a unipolar construct, it would be consequential for the employment of the RAS. According to Reise and Waller^63^, in the case of unipolar constructs, researchers often suggest rewriting the items included in the instrument that measure such constructs to elaborate items that provide information spread across the entire trait continuum, which is difficult to achieve. Finally, relationships and relationship satisfaction may be more affected by the absence of negative aspects of relationships than by the presence of diverse positive aspects of relationships^65^. Therefore, the RAS is a reliable measure of relationship satisfaction at low levels of the relationship satisfaction continuum that captures the presence of negativity in a relationship but not the absence of positivity in a relationship.

Regarding the validity of the Polish RAS, the results were satisfactory for the convergent, divergent, and criterion validity tests. These results were consistent with those of previous studies^10^. Furthermore, in line with past studies, we found that relationship satisfaction was negatively associated with impulsive behavior^12^ and was not related to fear of being single^10^. We also identified the lack of a link between workaholism and relationship satisfaction. Our results corroborate prior findings that showed the lack of a link between relationship adjustment and relationship satisfaction and workaholism^41^.

Finally, the concurrent validity of the Polish RAS was tested, and the results showed that relationship satisfaction was a strong predictor of the intent to continue the current relationship (in connection with a weak predictor of living vs. not living with a partner). Although various factors determine the stability of a relationship^66^, individuals in the current study who were more satisfied with their relationships were also more willing to continue their relationships, which suggests that they may also be less willing to consider terminating their relationships. This finding is consistent with literature showing the links between low relationship satisfaction and the risk of divorce/break-up^64^ and between higher relationship satisfaction and greater relationship stability^66^. Overall, these results verify the convergent, divergent, and criterion validity of the Polish RAS and provide a multilevel assessment of relationship satisfaction that can be applied to evaluate the links between relationship satisfaction and diverse outcomes, including mental and physical health, emotional and psychological well-being, depression and romantic loneliness.

### Limitations

The current analyses have both strengths and important limitations. The Polish target samples utilized in the current study were recruited via Facebook, so our sample was not representative of the entire Polish population. It excluded participants who do not use the internet or social media, including Facebook. Furthermore, we could not determine whether individuals who participated in the reference and validation samples 1 and 2 experienced higher or lower relationship satisfaction compared to participants who did not participate in the studies. Furthermore, the Polish reference and validation samples (and the Hungarian sample) included participants who were in nonmarital, engaged and marital relationships. Therefore, our results cannot be extended to individuals whose relational history includes the experience of divorce or widowhood and who have remarried or repartnered. The majority of participants in the Polish samples were childless, limiting the generalizability of our findings to parents. It is also important to note that the ratio of the Hungarian and Polish sample sizes to the U.S. sample size was not high. Since we utilized previously collected data in the USA, we could not increase the U.S. sample size to the required sample size of N > 500 for IRT analyses, which might affect the power of the IRT model^67^. In addition, our focal groups of individuals in formal and informal relationships did not reach the size of N > 500. Therefore, we encourage other researchers to obtain the required sample size for the focal groups when employing the IRT methodology. Finally, both the Hungarian and U.S. data and the Polish validation sample 2 utilized in this paper were collected on different occasions than the data collected for the Polish reference and validation sample 1. Notably, in Poland, the data in the reference and validation sample 1 were collected during the period of the COVID-19 pandemic. Thus, the possibility cannot be excluded that COVID-19-related distress might affect the obtained results because the COVID-19 pandemic produced adverse psychological consequences in the domain of mental health^68^. Furthermore, some recent studies have revealed that both men and women experienced decreased relationship satisfaction before and during the COVID-19 pandemic^69^.

## Supplementary Information

Supplementary Information.

## Data Availability

The raw and processed data collected in the Polish reference and validation samples 1 and 2, the data collected in the United States and the analysis code for this study are available at the Open Science Framework (OSF) repository (https://osf.io/7hv32/?view_only=53083d2e2869472490beece1b35b7294). The data collected in Hungary are available from the Hungarian authors by e-mail request (Fülöp et al., 2020). The runnable source code is available in the file Methods (Code) at the Open Science Framework (OSF) repository (https://osf.io/7hv32/?view_only=53083d2e2869472490beece1b35b7294). The materials used in the Polish reference sample and the links to the validation instruments in the Polish language are available in the file Materials at the Open Science Framework website (https://osf.io/7hv32/?view_only=53083d2e2869472490beece1b35b7294).
