# Supplementary material for: Psychometric functioning, measurement invariance, and external associations of the Relationship Assessment Scale in a sample of Polish Adults
Source: Sci Rep. 2022 Dec 22;12:22157. doi: 10.1038/s41598-022-26653-6 (PMC9773665; doi:10.1038/s41598-022-26653-6)
Supplement: Supplementary file 1 — Supplementary Information. [file 41598_2022_26653_MOESM1_ESM.docx]

| **Table S1**  *Reference and Validation Polish Samples Characteristics* | | | |
| --- | --- | --- | --- |
| Variable | Reference Sample  (n = 733) | Validation Sample 1  (n = 203) | Validation Sample 2  (n = 209) |
| Age, years |  |  |  |
| *M (SD)* | 32.70 (8.62) | 31.14 (7.56) | 24.91 (4.83) |
| Range | 18 - 75 | 19 - 54 | 19 – 46 |
| Gender, *n* (%) |  |  |  |
| Male | 317 (43.20%) | 42 (20.70%) | 72 (34.40%) |
| Female | 416 (56.80%) | 161 (79.30%) | 137 (65.50%) |
| Sexual orientation |  |  |  |
| Heterosexual | 602 (82.10%) | 164 (80.80%) | 196 (93.80%) |
| Homosexual | 50 (6.80%) | 10 (4.90%) | 3 (1.40%) |
| Bisexual | 73 (10.00%) | 25 (12.30%) | 8 (3.80%) |
| I do not know | 8 (1.10%) | 4 (2.00%) | 2 (1.00%) |
| Place of residence,  *n* (%) |  |  |  |
| Village | 55 (7.50%) | 15 (7.40%) |  |
| City < 25, 000 | 76 (10.40%) | 15 (7.40%) | - |
| City 25,000 - 50,000 | 68 (9.30%) | 14 (6.90%) | 21 (10.05%) |
| City 50,000 - 200,000 | 105 (14.30%) | 37 (18.20%) | 25 (11.96%) |
| City 200,000 - 500,000 | 331 (45.20%) | 122 (60.10%) | 23 (11.00%) |
| City > 500,000 | 98 (13.40%) | - | 140 (66.99%) |
| Highest education, *n* (%) |  |  |  |
| Primary school | - | - | 25 (12.00%) |
| Secondary education | 116 (15.80%) | 20 (9.90%) | 17 (8.10%) |
| High school | - | - | 63 (30.10%) |
| Higher education | 540 (73.70%) | 155 (76.40%) | - |
| Student | 77 (10.50%) | 28 (13.80%) | 104 (49.80%) |
| Type of relationships, *n* (%) |  |  |  |
| Non-marital relationships | 401 (54.70%) | 123 (60.60%) | 32 (15.30%) |
| Engaged relationships | 121 (16.50%) | 45 (22.20%) | 140 (67.00%) |
| Marriage | 211 (28.80%) | 35 (17.20%) | 37 (17.70%) |
| Cohabitation (Living with a partner) |  |  |  |
| Yes | 573 (78.20%) | 149 (73.40%) | 103 (49.30%) |
| No | 160 (21.80%) | 54 (26.60%) | 106 (50.70%) |
| Having a child/children |  |  |  |
| Yes | 116 (15.8%) | 35 (17.20%) | 26 (12.40%) |
| No | 617 (84.2%) | 168 (82.80%) | 183 (87.60%) |
| Duration of a relationship, years |  |  |  |
| *M (SD)* | 7.62 (7.28) | 5.98 (5.48) | 3.37 (3.50) |
| *Note.* The data in the calibration and validation samples 1 were collected in the scope of the current study, whereas the data in the validation sample 2 were collected in another project described in the papers by Adamczyk et al. (2021a, 2021b). | | | |

| **Table S2**  *The CFA Model Parameters Estimated in the Polish Reference Sample (n = 733)* |
| --- |

Latent Variables:
                  Estimate  Std.Err  z-value  P(>|z|)   Std.lv  Std.all
 Total =~                                                              
   RAS1    (.p1.)    1.917    0.090   21.297    0.000    1.917    0.887
   RAS2    (.p2.)    2.819    0.172   16.420    0.000    2.819    0.942
   RAS3    (.p3.)    1.722    0.086   19.911    0.000    1.722    0.865
   RAS4    (.p4.)    1.168    0.062   18.983    0.000    1.168    0.760
   RAS5    (.p5.)    1.510    0.074   20.309    0.000    1.510    0.834
   RAS6    (.p6.)    1.340    0.076   17.566    0.000    1.340    0.802
   RAS7    (.p7.)    0.774    0.042   18.271    0.000    0.774    0.612

Thresholds:
                  Estimate  Std.Err  z-value  P(>|z|)   Std.lv  Std.all
   RAS1|t1          -4.772    0.261  -18.310    0.000   -4.772   -2.207
   RAS1|t2          -3.831    0.183  -20.942    0.000   -3.831   -1.772
   RAS1|t3          -2.153    0.129  -16.681    0.000   -2.153   -0.996
   RAS1|t4           1.074    0.104   10.337    0.000    1.074    0.497
   RAS2|t1          -6.603    0.423  -15.596    0.000   -6.603   -2.207
   RAS2|t2          -4.946    0.290  -17.058    0.000   -4.946   -1.654
   RAS2|t3          -3.117    0.215  -14.517    0.000   -3.117   -1.042
   RAS2|t4           0.582    0.137    4.240    0.000    0.582    0.194
   RAS3|t1          -4.477    0.259  -17.290    0.000   -4.477   -2.248
   RAS3|t2          -3.668    0.185  -19.860    0.000   -3.668   -1.842
   RAS3|t3          -1.961    0.115  -17.098    0.000   -1.961   -0.985
   RAS3|t4           0.174    0.092    1.889    0.059    0.174    0.087
   RAS4|t1          -2.922    0.138  -21.238    0.000   -2.922   -1.901
   RAS4|t2          -2.155    0.103  -20.899    0.000   -2.155   -1.402
   RAS4|t3          -1.329    0.085  -15.706    0.000   -1.329   -0.864
   RAS4|t4          -0.155    0.072   -2.163    0.031   -0.155   -0.101
   RAS5|t1          -3.563    0.173  -20.605    0.000   -3.563   -1.968
   RAS5|t2          -2.754    0.131  -20.976    0.000   -2.754   -1.521
   RAS5|t3          -1.384    0.098  -14.159    0.000   -1.384   -0.764
   RAS5|t4           0.557    0.084    6.590    0.000    0.557    0.307
   RAS6|t1          -3.760    0.203  -18.524    0.000   -3.760   -2.248
   RAS6|t2          -3.112    0.150  -20.729    0.000   -3.112   -1.861
   RAS6|t3          -2.329    0.119  -19.634    0.000   -2.329   -1.393
   RAS6|t4          -1.004    0.090  -11.133    0.000   -1.004   -0.600
   RAS7|t1          -2.162    0.101  -21.312    0.000   -2.162   -1.710
   RAS7|t2          -1.469    0.076  -19.264    0.000   -1.469   -1.161
   RAS7|t3          -0.097    0.059   -1.658    0.097   -0.097   -0.077
   RAS7|t4           0.703    0.062   11.342    0.000    0.703    0.556

Variances:
                  Estimate  Std.Err  z-value  P(>|z|)   Std.lv  Std.all
  .RAS1              1.000                               1.000    0.214
  .RAS2              1.000                               1.000    0.112
  .RAS3              1.000                               1.000    0.252
  .RAS4              1.000                               1.000    0.423
  .RAS5              1.000                               1.000    0.305
  .RAS6              1.000                               1.000    0.358
  .RAS7              1.000                               1.000    0.625
   Total             1.000                               1.000    1.000

Scales y*:
                  Estimate  Std.Err  z-value  P(>|z|)   Std.lv  Std.all
   RAS1              0.463                               0.463    1.000
   RAS2              0.334                               0.334    1.000
   RAS3              0.502                               0.502    1.000
   RAS4              0.650                               0.650    1.000
   RAS5              0.552                               0.552    1.000
   RAS6              0.598                               0.598    1.000
   RAS7              0.791                               0.791    1.000

**Figure S1.**

*Test information curve for the seven items of the Polish RAS. n = 733. RAS = Relationship Assesment Scale.*


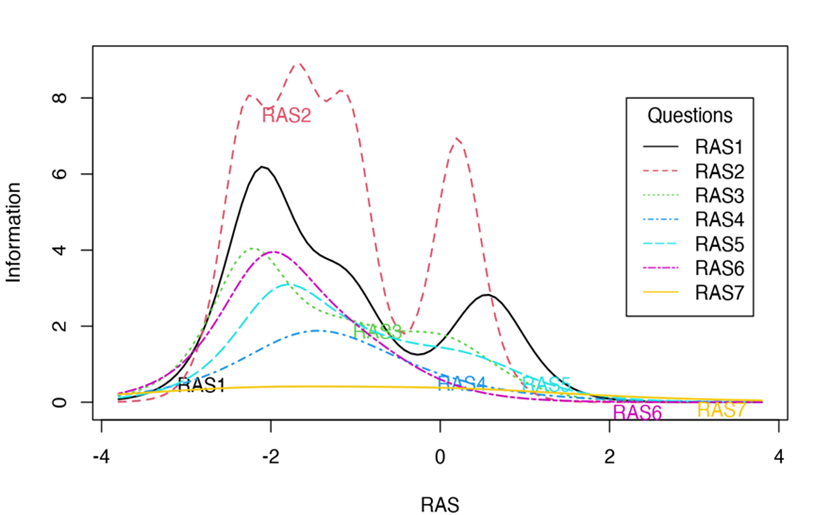


**
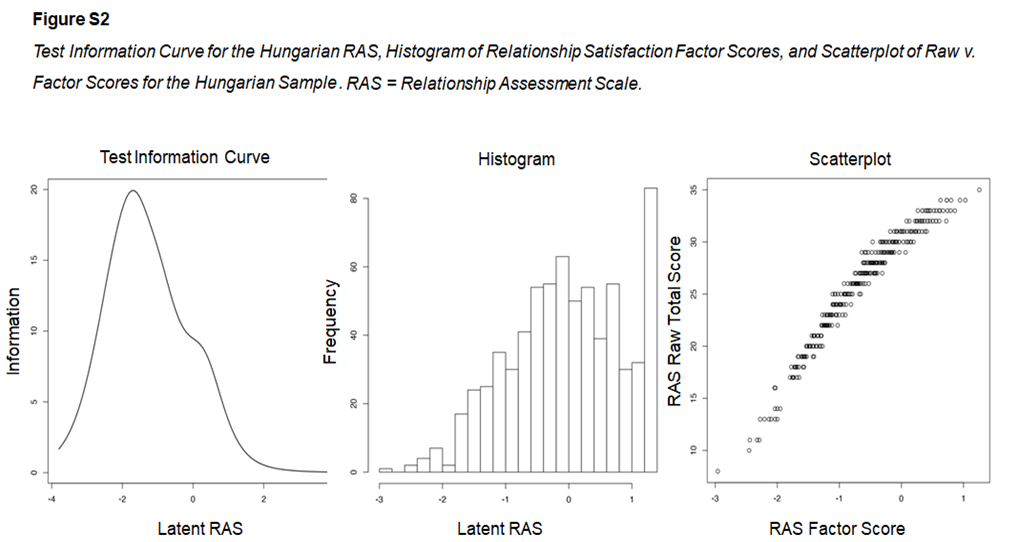
**

**
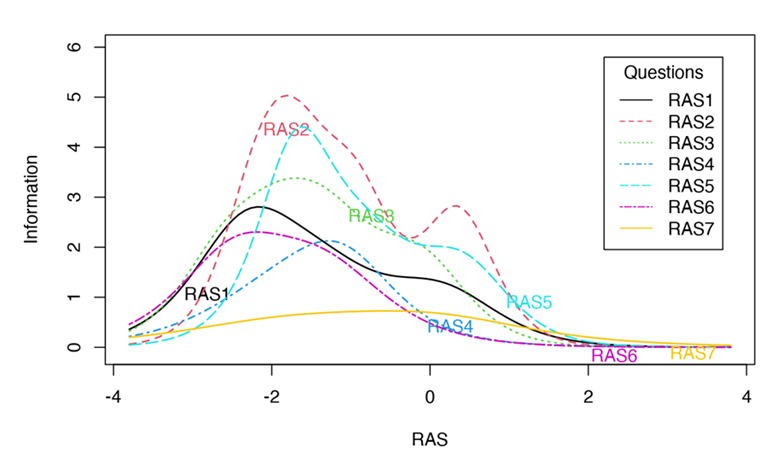
**

**Figure S3**

*Test information curve for the seven items of the Hungarian RAS. RAS = Relationship Assesment Scale.*

| **Table S3**  *Item Difficulty Location and Thresholds For Informational Curves in the Hungarian Sample* | | | | | | | | |
| --- | --- | --- | --- | --- | --- | --- | --- | --- |
| Items | a | SE | Threshold 1 | Threshold 2 | Threshold 3 | Threshold 4 | Information in range  [-3, 3] SD | Proportion of information in range vs Total information |
| RAS1 | 2.078 | 0.179 | -2.631 | -2.112 | -1.257 | 0.165 | 7.474 | .8991 |
| RAS2 | 3.256 | 0.307 | -2.224 | -1.651 | -0.904 | 0.366 | 12.774 | .9810 |
| RAS3 | 2.541 | 0.241 | -2.651 | -1.861 | -1.165 | -0.087 | 9.353 | .9201 |
| RAS4 | 1.278 | 0.123 | -2.175 | -1.883 | -0.686 | -1.364 | 4.635 | .9067 |
| RAS5 | 2.529 | 0.233 | -1.849 | -1.590 | -0.767 | 0.424 | 9.974 | .9860 |
| RAS6 | 1.602 | 0.161 | -2.685 | -2.449 | -1.509 | -1.009 | 5.401 | .8429 |
| RAS7 | 0.864 | 0.079 | -2.250 | -1.666 | 0.114 | 0.309 | 2.915 | .8436 |
| *Note.* n = 703; a = discrimination parameter estimates in RAS; Threshold 1 = difficulty parameter estimates between response categories 0 and 1; Threshold 2 = difficulty parameter estimates between response categories 1 and 2; Threshold 3 = difficulty parameter estimates between response categories 2 and 3; Threshold 4 = difficulty parameter estimates between response categories 3 and 4.  Area under curve with range [-3, 3] SD raw and percent, compared to total result of information function. | | | | | | | | |

| **Table S4**  *Regression Analysis Predicting Intent to Continue the Current Relationships From Demographics and the Polish RAS* | | | | | | | | |
| --- | --- | --- | --- | --- | --- | --- | --- | --- |
| **Coefficients^a^** | | | | | | | | |
| Model | | Unstandardized  Coefficients | | Standardized  Coefficients | *t* | *p*-value | 95% Confidence Interval for B | |
|  |  | B | Stand. Error | Beta |  |  | Lower Bound | Upper Bound |
| 1 | Constant | 2,476 | ,229 |  | 10,802 | ,000 | 2,024 | 2,929 |
|  | Age | -,001 | ,007 | -,010 | -,104 | ,917 | -,014 | ,013 |
|  | Gender | ,014 | ,091 | ,011 | ,153 | ,879 | -,165 | ,193 |
|  | Type of a relationship | ,095 | ,056 | ,148 | 1,682 | ,094 | -,016 | ,206 |
|  | Living with a partner | ,442 | ,109 | **,317** | **4,054** | **,000** | ,227 | ,658 |
|  | Duration of living with a partner | -,001 | ,001 | -,076 | -,487 | ,627 | -,003 | ,002 |
|  | Duration of a relationship | -,001 | ,001 | -,096 | -,678 | ,499 | -,003 | ,001 |
|  | Having children | -,094 | ,113 | -,074 | -,832 | ,406 | -,318 | ,129 |
| 2 | Constant | ,399 | ,252 |  | 1,579 | ,116 | -,100 | ,897 |
|  | Age | ,001 | ,005 | ,020 | ,269 | ,788 | -,009 | ,012 |
|  | Gender | -,001 | ,068 | -,001 | -,019 | ,985 | -,136 | ,134 |
|  | Type of a relationship | ,049 | ,043 | ,076 | 1,147 | ,253 | -,035 | ,133 |
|  | Living with a partner | ,226 | ,085 | **,162** | **2,675** | **,008** | ,059 | ,393 |
|  | Duration of living with a partner | ,000 | ,001 | -,069 | -,587 | ,558 | -,002 | ,001 |
|  | Duration of a relationship | ,000 | ,001 | -,015 | -,136 | ,892 | -,002 | ,001 |
|  | Having children | ,056 | ,087 | ,044 | ,651 | ,516 | -,115 | ,227 |
|  | RAS Relationship satisfaction | ,535 | ,047 | **,653** | **11,312** | **,000** | ,441 | ,628 |
| a. Dependent variable: Intent to continue the current relationship in the future. | | | | | | | | |

**Materials used in Reference Sample and Validation Samples 1 and 2**

**The Relationship Assessment Scale (RAS; Hendrick, 1988)**

**Polish translation by Monfort et al. (2014)**

Zakreśl literę wskazującą odpowiedź, która najlepiej oddaje Twoje przekonanie na dany temat:

**Jak bardzo Twój partner/partnerka odpowiada Twoim potrzebom?**

A B C D E

Słabo Przeciętnie Nadzwyczaj

**Ogólnie rzecz biorąc, jak bardzo jesteś zadowolony/a ze swojego związku?**

A B C D E

Niezadowolony/a Przeciętnie Niezmiernie zadowolony

**Jak udana jest Twoja relacja w porównaniu z większością relacji?**

A B C D E

Słaba Przeciętna Doskonała

**Jak często zdarza Ci się myśleć, że lepiej byłoby nie wchodzić w ten związek?**

A B C D E

Nigdy Przeciętnie Bardzo często

**W jakim stopniu ten związek spełnił Twoje pierwotne oczekiwania?**

A B C D E

Prawie w ogóle Przeciętnie Całkowicie

**Jak bardzo kochasz swojego partnera/partnerkę?**

A B C D E

Niezbyt Przeciętnie Bardzo mocno

**Jak dużo problemów istnieje w waszym związku?**

A B C D E

Bardzo mało Przeciętnie Bardzo dużo

References:

Monfort, S. S., Kaczmarek, L. D., Kashdan, T. B., Drążkowski, D., Kosakowski, M., Guzik, P., Krauze, T., Gracanin, A. (2014). Capitalizing on the success of romantic partners: laboratory investigation on subjective, facial, and physiological emotional processing. *Personality and Individual Differences, 68*, 149-153. doi: <http://dx.doi.org/10.1016/j.paid.2014.04.028>

The paper can be retrieved from:
https://www.sciencedirect.com/science/article/abs/pii/S0191886914002669

**Materials used in Reference and Validation Sample 1**

**Kwestionariusz Danych Osobowych [Personal Information Questionnaire]**

Uprzejmie prosimy o zaznaczenie swojej odpowiedzi, wybierając jedną z podanych opcji lub wpisując informacje w miejscach, które tego wymagają.

1. Wiek ….........
2. Płeć

[ ] kobieta

[ ] mężczyzna

[] inne

1. Miejsce zamieszkania:

[ ] wieś

[ ] miasteczko do 25 000 mieszkańców

[ ] miasto od 25 000 do 50 000 mieszkańców

[ ] miasto od 50 000 do 200 000 mieszkańców

[ ] miasto od 200 000 do 500 000 mieszkańców

1. Wykształcenie

[ ] podstawowe

[ ] gimnazjalne

[ ] zasadnicze zawodowe

[ ] średnie

[ ] wyższe

[ ] student

1. Czy aktualnie posiada Pan/Pani partnera życiowego/partnerkę życiową?

[ ] tak

[ ] nie

1. Które określenie (tylko jedno) odnosi się do Pana/Pani?

[ ] związek nieformalny (związek, który nie jest małżeństwem)

[ ] narzeczeństwo

[ ] żonaty/zamężna

[ ] w separacji

[ ] rozwiedziony/rozwiedziona

[ ] wdowiec/wdowa

1. Od jak dawna jest Pan/Pani z obecną partnerką/partnerem?

Prosimy o podanie czasu, wskazując, czy dana liczba odnosi się do lat, czy też miesięcy, np. 5 lat; 6 miesięcy................. …..

8. Czy aktualnie mieszka Pani/Pan ze swoim partnerem/partnerką?

[ ] Tak

[ ] Nie

9. Od jak dawna mieszka Pani/Pan Pana/Pani z obecną partnerką/partnerem?

Prosimy o podanie czasu, wskazując, czy dana liczna odnosi się do lat, czy też miesięcy, np. 5 lat; 6 miesięcy

……………………………….

10. Czy chciałby Pan/chciałby Pani kontynuować w przyszłości aktualny związek? Proszę odpowiedzieć na to pytanie, posługując się skalą od 0 do 3:

0 – wcale/w ogóle nie chciałbym/chciałabym

1 - trochę

2 – do pewnego stopnia

3 – bardzo bym chciał/chciała

11. Orientacja seksualna:

[ ] heteroseksualna

[ ] homoseksualna

[ ] biseksualna

[ ] nie wiem

12. Czy ma Pan/Pani dzieci?

[ ] tak

[ ] nie

**Materials used in Reference and Validation Sample 1**

**Personal Information Questionnaire**

Please indicate your response by selecting one of the options provided or by typing the information in the spaces provided.

1. Age ............

2. Gender

[ ] female
[ ] male
[ ] other

3. Place of residence:

[ ] rural
[ ] town of up to 25,000 inhabitants
[ ] town of 25,000 to 50,000 inhabitants
[ ] town of 50,000 to 200,000 inhabitants
[ ] city of 200,000 to 500,000 inhabitants

4. Education:

[ ] elementary education
[ ] lower secondary school
[ ] basic vocational education
[ ] secondary education
[ ] higher education
[ ] student

5. Do you currently have a life partner?

[ ] yes
[ ] no

6. Which term (only one) applies to you?

[ ] informal relationship (relationship that is not marriage)
[ ] engaged
[ ] married
[ ] separated
[ ] divorced
[ ] widowed

7. How long have you been with your current partner?
Please indicate the length of time, indicating whether the number refers to years or months, e.g. 5 years; 6 months.

.........................

8. Are you currently living with your partner?

[ ] yes

[ ] no

9. How long have you been living with your current partner?

Please specify the length of time, indicating whether the number refers to years or months, e.g. 5 years; 6 months

.....................................

10. Would you like to continue your current relationship in the future? Please answer this question using a scale from 0 to 3:

0 - not at all

1 - a little

2 - to some extent

3 - very much

11. Sexual Orientation:

0 = heterosexual

1 = homosexual

2 = bisexual

3= don’t know

12. Do you have children?

1 = yes

0 = no

**Additional Materials Used in Validation Sample 1**

**The Couples Satisfaction Index (CSI; Funk, Rogge, 2007)**

**Polish adaptation by Stawska (2011)**

***The Couples Satisfaction Index*** (CSI; Funk, Rogge, 2007) (Polish adaptation – Stawska, 2011) is a tool to measure relationship satisfaction. In the validation sample 1 we used a 4-item version of the CSI. Participants are asked to rate each statement using a Likert scale of different response format. The higher the score indicates higher relationship satisfaction. In the current sample, McDonald's omega was .90.

The Polish version can be retrieved from:

https://www.researchgate.net/publication/274009543_Skala_satysfakcji_ze_zwiazku_CSI-32_Funk_Rogge_2007_polska_adaptacja_Stawska_2011

References:
Stawska, M. (2011). Skala satysfakcji ze związku CSI-32 [Couples Satisfaction Index – 32].

**The Relationship Satisfaction Status Scale (ReSta; Lehmann et al, 2015)**

**Polish adaptation by Adamczyk (2019).**

***The Satisfaction with Relationship Status Scale*** (ReSta; Lehmann et al. 2015) (Polish adaptation - Adamczyk (2019) was designed to measure satisfaction with relationship status. It consists of five questions rated on a 4-point Likert scale from 0 (*not at all)* to 3 (*to a great extent*). In the current sample, McDonald's omega was .95.

The Polish adaptation is described and available in:

Adamczyk, K. (2019). Development and validation of a Polish-language version of the Satisfaction with Relationship Status Scale (ReSta). *Current Psychology*, *38*(1), 8-20. https://doi.org/10.1007/s12144-017-9585-9

The paper can be retrieved from:

https://link.springer.com/article/10.1007/s12144-017-9585-9

**The Quality of Relationships Inventory (QRI; Pierce et al., 1991)**

**Polish adaptation by Suwalska-Braancewicz et al. (2015)**

**The Quality of Relationships Inventory** (QRI; Pierce et al., 1991) (Polish adaptation - Suwalska-Barancewicz et al., 2015) assesses the relationship quality. It consists of 25 items that create the following three subscales: Perceived Support, Relationship Depth Scale and Conflict. Participants rate statements using a 4-point Likert scale ranging from *1* (never/ to no extent/ to no extent) to *4* (always/ to every extent/ to a very high extent). In this current sample, the coefficient of McDonald’s omega was .84 for Perceived Support, .71 Relationship Depth Scale and .86 Conflict, respectively.

The Polish adaptation is described in:

Suwalska-Barancewicz, D. K., Liberska, H., & Izdebski, P. K. (2015). Inwentarz Jakości Związku – polska adaptacja [The Quality of Relationships Inventory–Polish adaptation]. *Psychologia Rozwojowa, 1*, 91-105. doi:10.4467/20843879PR.15.006.3478

The paper can be retrieved from: https://www.ejournals.eu/Psychologia-Rozwojowa/2015/Numer-1-2015/art/4978/

**The Fear of Being Single Scale (FBSS; Spielmann et al., 2013)**

**Polish adaptation by Adamczyk et al. (2021)**

*The Fear of Being Single Scale* (FBSS; Spielmann et al., 2013) (Polish adaptation - Adamczyk et al., 2021) is a scale used to measure the fear of being single. The scale consists of six items rated on a 5-point Likert scale from *1* ( completely false) to *5* (completely true). In the current sample coefficient of McDonald’s omega was .86.

The Polish adaptation is described in:

Adamczyk, K., Trepanowski, R., Celejewska, A. et al. (2021). The Polish adaptation and further validation of the Fear of Being Single Scale (FBSS). *Current Psychology,* 40, 2499–2509. doi: https://doi.org/10.1007/s12144-019-00192-2

The paper can be retrieved from:

https://link.springer.com/article/10.1007/s12144-019-00192-2#citeas

**Workaholism Battery (Spence & Robbins, 1992)**

**Polish adaptation by Malinowska et al. (2010)**

**Workaholism Battery Scale** (WorkBAT; Spence, Robbins 1992) (Polish adaptation - Malinowska et al., 2010) was designed to measure work addiction. It consists of 15 statements and includes three subscales: Feeling driven to work, Job Enjoyment and Work Involvement. Participants rate the statements on a 5-point Likert scale from *1* (strongly agree) to *5* (strongly disagree). Due to the low reliability of the Work Involvement subscale, the two-factor model consisting of Work Involvement/Feeling driven to work (McDonald’s ω = .78) and Enjoyment (McDonald’s ω = .84) subscale was used in the current sample.

The Polish adaptation is described in:

Malinowska, D., Tokarz, A., & Gad, N. (2010). Wstępne badania nad adaptacją skali Workaholism Battery (WorkBAT) Spence i Robbins [A preliminary adaptation study of Spence and Robbins’ Workaholism Battery (WorkBAT)]. *Studia Psychologiczne*, *48*(3), 35–42.

The paper can be retrieved from: http://cejsh.icm.edu.pl/cejsh/element/bwmeta1.element.doi-10_2478_ppb-2014-0027

**The Impulsive Behavior Scale Short Version (SUPPS-P; Cyders et al., 2015)**

**Polish adaptation by Poprawa (2019**)

***The Impulsive Behavior Scale Short Version*** (SUPPS-P; Cyders et al., 2014) (Polish adaptation – Poprawa, 2019) is a tool measuring . In the current sample, the 4-item subscale assessing impulsive behavior was used. The statements are rated using a 4-point Likert scale from *1* (completely agree) to *4* (completely disagree). In this sample, McDonald’s  *ω* was .85.

The Polish adaptation is described at:

Poprawa, R. (2019). Badania nad polską skróconą wersją Skali Impulsywnego Zachowania UPPS-P [Research on the Polish short version of the Impulsive Behavior Scale UPPS-P]. *Alcoholism and Drug Addiction/Alkoholizm i Narkomania, 32*(1), 35-62. <https://doi.org/10.5114/ain.2019.85767>

The paper can be retrieved from:

https://www.termedia.pl/Badania-nad-polska-skrocona-wersja-Skali-Impulsywnego-Zachowania-UPPS-P,117,36870,0,0.html

**Materials used in Validation Sample 2 Described in a Paper by Adamczyk et al. (2021b)**

**Mental Health Continuum - Short Form (Keyes, 2002)**

**Polish adaptation by Karaś et al. (2014)**

***The 14-item Mental Health Continuum-Short Form*** (MHC – SF; Keyes, 2002) and its Polish adaptation (Karaś et al., 2014) were used to assess emotional and psychological well-being. This questionnaire includes 14 items assessing emotional, psychological, and social well-being, and in the current analyses we used two subscales measuring emotional and psychological well-being. Participants rated questions about how they have been feeling during the past month using a scale ranging from 0 (*never*) to 5 (*every day*). In this sample, McDonald’s  *ω*  was .91 for emotional well-being and .89 for psychological well-being.

The Polish adaptation is described in:

Karaś, D., Cieciuch, J., & Keyes, C. L. M. (2014). The Polish adaptation of the Mental Health Continuum-Short Form (MHC-SF). Personality and Individual Differences, 69, 104–109. [https://doi.org/10.1016/j.paid.2014.05.011](https://psycnet.apa.org/doi/10.1016/j.paid.2014.05.011)

The paper can be retrieved from:

<https://www.sciencedirect.com/science/article/abs/pii/S0191886914002955>

**The Centre for Epidemiological Studies – Depression Scale** **(CES-D; Radloff, 1977)**

**Polish adaptation by Kaniasty (2003).**

Depressive symptoms were measured using the Center for Epidemiological Studies Depression Scale (CES-D; Radloff, 1977) (Polish adaptation – Kaniasty, 2003). The questionnaire consists of 20 statements measuring the frequency of depressive symptoms including depressive affect (7 items), absence of well-being (4 items), somatic symptoms (7 items), and interpersonal affect (2 items). Participants indicate the frequency of depressive symptoms in the past week using a four-point scale (0 = rarely, or not at all to 3 = most of the time or all the time). In this sample, McDonald’s *ω* was .93.

The Polish adaptation is described in:

Kaniasty, K. (2003). *Klęska żywiołowa czy katastrofa społeczna? Psychospołeczne konsekwencje polskiej powodzi 1997 roku.* [Natural or social catastrophy? Psychosocial consequences of 1997 flood in Poland]. Gdańsk: Gdańskie Wydawnictwo Psychologiczne.

**SF-12v2 (Maruish, 2012)**

**The Polish version of the SF-12v2 was obtained and used in the scope of the purchased license**

To assess physical and mental health, participants completed the SF-12v2 which is as the SF-12 Health Survey (SF-12; Ware et al., 1995) a generic measure of health status (Maruish, 2012). The SF-12v2 includes a physical and mental component summary (PCS and MCS) measure. The SF-12v2 Health Survey in English and Polish language versions is consisted of12 items, and participants are asked to provide answers using a 5-point Likert scale from
1 to 5 in different wording (with the exception of the item. no. 3 using a 3-point Likert scale). In this sample, McDonald’s *ω* was .87 for mental health component and .77 for physical component.

References:

Maruish, M. E. (Ed.). (2012). *User’s manual for the SF-12v2 Health Survey* (3rd ed.). Lincoln, RI: Quality Metric Incorporated.

**The Social and Emotional Loneliness Scale for Adults (SELSA-S) (DiTommaso et al., 2004)**

**Polish adaptation by Adamczyk & DiTommaso (2014)**

Romantic loneliness was measured with the 5-item romantic loneliness subscale from the Social and Emotional Loneliness Scale for Adults - Short Form (SELSA-S; DiTommaso et al., 2004) (Polish adaptation - Adamczyk & DiTommaso, 2014). Participants were asked to respond to statements using a 7-point Likert-type scale ranging from 1 (*strongly disagree*) to 7 (*strongly agree*). In this sample, McDonald’s *ω* was .81.

The Polish adaptation is described in:

Adamczyk, K., & DiTommaso, E. (2014). Psychometric properties of the Polish version of the Social and Emotional Loneliness Scale for Adults (SELSA-S). *Psychological Topics, 23*(3), 327-341. UDK – 159.923.33.072-057.875(438)
